# Anti-M Antibody in Blood Transfusion: An Underestimated Factor in Service Delays and Expenditures

**DOI:** 10.7759/cureus.85004

**Published:** 2025-05-28

**Authors:** Suman S Routray, Nirupama Sahoo, Biswajit Bhuyan, Sukanta Tripathy, Gopal K Ray, Devi Acharya, Abhra Barman

**Affiliations:** 1 Department of Immunohaematology and Blood Transfusion, Kalinga Institute of Medical Sciences, Bhubaneswar, IND; 2 Department of Haematology, Kalinga Institute of Medical Sciences, Bhubaneswar, IND; 3 Transfusion Medicine, All India Institute of Medical Sciences, Guwahati, Guwahati, IND

**Keywords:** alloantibody, blood group discrepancy, crossmatch incompatibility, mns blood group system, m-variant, pretransfusion testing, turnaround time

## Abstract

Background and objective

The effect of anti-M (IgM or IgG) on transfusion services is hardly addressed in the literature. Here, the prevalence of clinically significant anti-M among the donor population and patients and their impact on turnaround time for the issue of blood units and cost was analysed.

Methods

A retrospective review of immunohaematological records from blood donors and patient electronic medical records at a tertiary care hospital, Odisha, India over a two-year period was conducted to assess the impact of anti-M antibody developed in donors or patients on blood transfusion services.

Results

Anti-M antibodies were identified in approximately one in every 7,155 blood donations and in one in 2,380 patients (20 out of 47,613 transfusion requests). These antibodies were detected across all patient age groups (ranging from four to 80 years) and in four young donors aged 18 to 30 years. Among the identified cases, the anti-M antibody was of the IgM + IgG type in three donors and 13 patients.

Resolving blood group discrepancies due to anti-M required an average of eight hours and necessitated the expertise of a certified immunohematology (IH) technician, as well as advanced testing procedures. This led to a statistically significant increase (p < 0.05) in testing costs - approximately 10-fold higher than routine testing. The median turnaround time for issuing compatible blood units increased significantly (Median: 24 hours, p < 0.05) for hospitalized patients. Two cases with anti-M were suspected to have the M variant with the M-positive phenotype, not degraded by papain treatment. Twenty units were found to be M-negative after typing 247 units for 13 patients. The scheduled surgical procedure was delayed by a median of two days (range one to eight days) in eight cases to make an M-negative compatible blood unit available. These delays further escalated pretransfusion testing costs and extended hospital stays. In one instance, an M-negative unit could not be found even after testing 48 units, necessitating referral to another centre.

Conclusion

The presence of clinically significant anti-M antibodies in our population resulted in transfusion and surgical start time delay, a 10-fold increase in cost for pretransfusion testing. Establishing advanced centres with an M-negative donor database and promoting inter-centre collaboration could help address the challenges of finding the required number of M-negative units.

## Introduction

The MNS blood group system is highly polymorphic, with approximately 88.7% of the Indian population expressing the M antigen [1]. Anti-M is a naturally occurring, cold-reacting IgM antibody and is typically found in individuals who lack the M antigen. It exhibits a dosage effect and does not agglutinate papain- or ficin-treated red blood cells due to the enzyme sensitivity of the M antigen. Most cases of anti-M are clinically insignificant and primarily cause blood grouping discrepancies due to their reactivity at room temperature (22-24°C) [2]. However, the presence of an IgG component, particularly those reactive at 37°C and in the anti-human globulin (AHG) phase, may be clinically significant. These antibodies can cause haemolytic transfusion reactions (HTRs), decreased red cell survival, and hemolytic disease of the foetus and newborn (HDFN) [3,4].

While anti-M has been more commonly observed in pregnant women [3], data on its prevalence in the general population, particularly among blood donors and patients admitted to hospitals for transfusion, remains limited. Much of the evidence depends on case reports and not a systematic review of its effect on pre-transfusion testing and transfusion practice [5]. Anti-M in blood donors can complicate ABO blood group typing, causing unexpected reactions in reverse grouping, and may lead to delayed resolution [6,7]. Similarly, in transfusion-dependent patients, clinically significant anti-M may necessitate antigen-negative red cell selection. This requirement involves extended antibody identification procedures, increased laboratory workload, repeated cross-matching, and the sourcing of rare phenotype units [8]. Collectively, these factors can elevate the overall cost of transfusion support and contribute to delays in patient care. Despite these challenges, the full extent of anti-M's impact on transfusion services remains underreported and is frequently underestimated in routine clinical practice. Consequently, many surgeons may not fully appreciate the time, resources, and complexity involved in securing compatible blood for patients with alloantibodies [9].

This study aims to evaluate the operational and economic implications of anti-M antibody detection within transfusion services. By analysing delays in blood issuance and the associated healthcare costs, the study seeks to underscore the need for increased awareness, standardized protocols, and potential policy interventions to alleviate the burden posed by this often underestimated alloantibody.

## Materials and methods

Study design and population

This retrospective study was conducted in the Department of Transfusion Medicine at a tertiary care referral hospital, covering the period from January 2023 to December 2024. The study aimed to analyze cases involving anti-M antibodies detected in blood donors and patients undergoing pre-transfusion testing. This study was conducted in accordance with institutional guidelines for research involving retrospective data and was approved by the Institutional Ethics Committee (IEC) of Kalinga Institute of Industrial Technology (KIIT) University (approval no: KIIT/KIMS/IEC/2025/2025, dated March 25, 2025).

Data collection

Case records were retrieved from paper-based immunohematology records, and additional clinical data were extracted from electronic medical records. The study included all cases where anti-M was identified during routine pre-transfusion testing. Key data points included patient demographics, transfusion history, blood grouping discrepancies, crossmatch incompatibilities, and transfusion-related adverse events. Cases with incomplete records were excluded from the study. 

Additionally, the impact of anti-M on transfusion service workflow, including resource utilization (consumables, manpower, time for resolution) and cost, was assessed. The cost analysis included both direct and indirect costs, such as laboratory resources, additional immunohematology (IH) testing, and delays in transfusion.

Detection and characterization of anti-M

**Figure 1:**
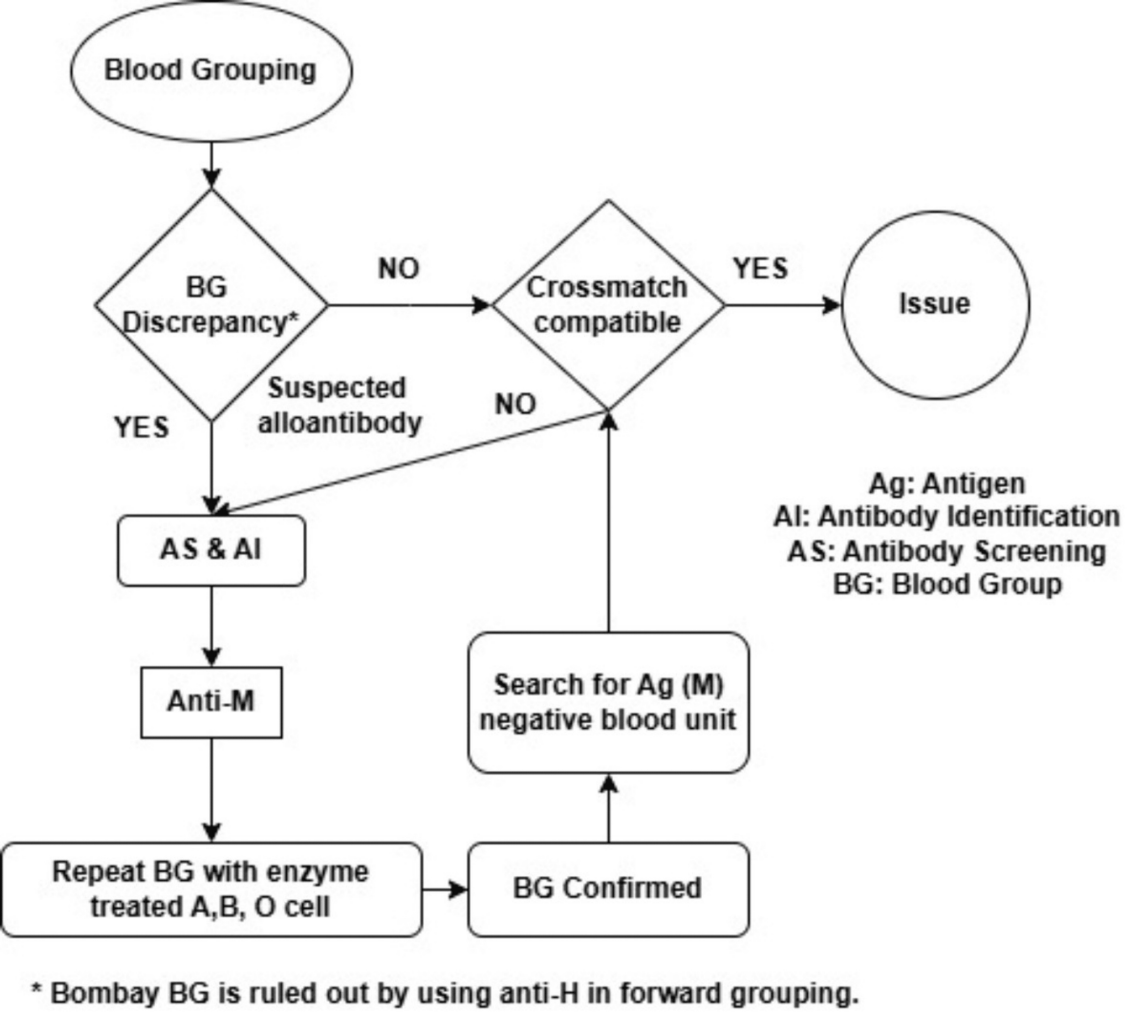
Flowchart of the immunohematology (IH) workup for anti-M Ag: antigen, AI: antibody identification, AS: antibody screening, BG: blood group Image Credit: G K Ray

All blood donors and patients underwent routine irregular antibody screening as part of standard IH protocols. The presence of anti-M was identified during the investigation of blood group discrepancies or crossmatch incompatibilities as shown in Figure 1.

Resolution of Blood Group Discrepancies

Unexpected additional reactions observed during reverse grouping with A1, B, and O reagent cells prompted further investigation using advanced immunohematological techniques. This included the use of commercially available lectins. Antibody screening (ReaCell I-II-III, Tulip Diagnostics Pvt. Ltd., Bambolim, India) and identification panels (ReaCell Panel, Tulip Diagnostics Pvt. Ltd.) were performed at room temperature (22-24°C). Enzyme-treated A1 and B cells (papain-treated, Tulip Diagnostics Pvt. Ltd.) were employed to aid in the resolution of these discrepancies.

Crossmatch-Incompatibility Workup

Antibody identification was performed in the AHG phase using commercial cell panels. Advanced methods, including selected cells and enzyme-treated panels, were used where necessary.

Clinical Significance Determination

Anti-M was classified as clinically significant if reactive at 37°C or in the AHG phase. Differentiation between IgM and IgG components was performed using dithiothreitol (DTT) treatment. Murine monoclonal anti-M antisera (Immucor Inc., Norcross, GA, USA) was used for red cell antigen phenotyping to assess M antigen status in both donors and patients.

Transfusion management and outcome assessment

For patients requiring transfusion, M antigen-negative packed red cell (PRC) units were selected and cross-matched using the Coombs gel card method (Matrix Gel Card, Tulip Diagnostics Pvt. Ltd.).

Patient outcomes, including transfusion success, delays in scheduled surgeries, extended hospital stays, and any transfusion reactions, were documented.

The resource burden was estimated based on the number of consumables used, additional manpower required, and cost incurred for extended pre-transfusion testing.

Cost Analysis

Regarding additional cost for IH workup, the standard cost for routine blood grouping and crossmatching is approximately 300 in Indian rupees (INR). However, in cases involving anti-M antibodies, the associated expenses are considerably higher. Antibody screening and identification procedures cost approximately INR 1,500 per test. Furthermore, locating an M antigen-negative blood unit incurs an additional cost of around INR 150 per unit.

Definitions

Turnaround Time (TAT)

TAT is the time taken to complete crossmatching for a single unit of blood, measured from the moment the blood requisition and sample are received. The average turnaround time at our blood center is calculated as the sum of the turnaround times for all crossmatched blood units divided by the total number of units crossmatched in a year. For routine crossmatching, the average TAT is 70 minutes.

Surgical Start Time Delay

This is the delay, measured in hours or days, between the scheduled and actual start time or date of surgery, attributable to the unavailability of M antigen-negative blood units. This includes any additional days required to obtain the next available operating theatre (OT) slot for the surgeon.

Statistical analysis

The data were analyzed using SPSS Statistics version 21 (IBM Corp., Armonk, NY, USA). Descriptive statistics were used to analyze the prevalence and characteristics of anti-M antibodies. Median was used to measure the central tendency of the given set of data and the Mann-Whitney U test was used to compare the median values. P value of < 0.05 is considered significant.

## Results

Prevalence and characteristics of anti-M in blood donors and patients

Among 28,620 blood donors screened for irregular antibodies, nine cases tested positive, of which four (44.4%) were identified as anti-M. This corresponds to an estimated prevalence of one in 7,155 donations. All anti-M-positive donors were young adult males (aged 18-30 years) with no prior history of transfusion.

A total of 195 cases with positive antibody screening were identified from 47613 blood requests, of which 20 (10.3%) were confirmed as anti-M. Twelve patients were female, and out of these 12, eight cases were in the reproductive age group (15-49 years). The age range of affected patients was four to 80 years. All the demographic details and characteristics of anti-M are depicted in Table 1. Patient details are provided in the supplementary table (Appendix 1).

**Table 1:** Demography details, transfusion history, and characteristics of anti-M in donor and patient population Pos: Positive; Neg: Negative; IQR: Interquartile Range ^1^Individual donor ages: 23, 25, 29, 30 *All patients are with anemia (sickle cell disease, cirrhosis of liver, cerebrovascular accident, acute pancreatitis, decompensated chronic liver disease) **Surgical cases belongs from department of general surgery (n=4), obstetrics and gynaecology (n=3), orthopedics (n=3), neurosugery (n=1), cardiothoracic vascular surgery (n=1), urology (n=1) and ear, nose and throat (n=2).

Donor/Patient characteristics	Donor (n=04)	Patient (n=20)
Age (in years)	^1^Median: 27 (IQR: 24.5-29.25)	Median: 42.5 (IQR: 28.5-65.75)
Sex	All male	Male: 08 Female:12 (Reproductive age group:08)
Transfusion History No transfusion	04	15
With Transfusion	00	05
Donor/Patient type	Voluntary:02 (Repeat:01) Replacement: 02 (Repeat:02)	Medical: 05* Surgical: 15**
BloodGroup A pos	00	05
B Pos	00	05
O Pos	03	09
AB Pos	01	01
Neg(if any)	00	00
Anti-M Type IgM	01	01
IgG	00	06
IgM+IgG	03	13
M Phenotype Negative	04	18
Positive	00	02 (Resistant to enzyme)

Blood group discrepancies, presenting as unexpected extra reactions in reverse grouping with A1, B, and O reagent cells, were observed in all four donors and 14 patients, while crossmatch incompatibility was observed in all patients except one case. Further IH workup confirmed the presence of anti-M with both IgM and IgG components in 16 cases (three donors and 13 patients). None of the donors were previously aware of their anti-M status.

All the donors and 18 patients, out of 20 patients, were found to be M antigen negative. However, two cases (Case 5 and Case 6) demonstrated an unusual serologic pattern-reacting as M-positive with monoclonal anti-M antisera but negative with polyclonal anti-M, suggesting a modified M antigen variant resistant to enzyme degradation. Anti-M was identified across all ABO blood groups, with the highest prevalence in O group patients.

Anti-M was identified during pre-operative pre-transfusion testing for these 20 patients (five medical and 15 surgical patients). The antibody exhibited reactivity at both room temperature and in the AHG phase, with no agglutination upon enzyme treatment, confirming anti-M specificity. Anti-M demonstrated a dosage effect in all cases, reacting more strongly with M+N- red cells than with M+N+ cells. Regarding immunoglobulin class distribution, 16 (65%) cases exhibited both IgG and IgM components, six (30%) had IgG alone, and only two (5%) had IgM alone. Clinically significant anti-M (reactive at 37°C or in the AHG phase) was identified in 19 (95%) out of 20 cases.

Impact of anti-M on transfusion services

The impact of anti-M on transfusion services is depicted in Table 2. Resolution of blood group discrepancy required dedicated IH-trained personnel, with the process taking a median of 480 (IQR: 477-522) minutes which was significantly higher (p<0.05) from the routine blood grouping with median duration of 34 (IQR: 32-40.5) minutes by gel technology. The additional cost incurred per case for blood grouping was about INR 1730. Since fresh frozen plasma (FFP) from these donors contained irregular antibodies, it was discarded, while PRC were processed for patient use. All affected donors were informed and counselled regarding their irregular antibody status and its implications for future donations. 

**Table 2:** Impact of anti-M on transfusion service BT: Blood Transfusion; INR: Indian Rupees; OT: Operation Theatre; IQR: Interquartile range ^1^Patients with anti-M represent 13 cases who required blood transfusions ^2^Routine cases comprised 40 cases without any blood group discrepancies and alloantibodies, requiring blood transfusions ^3^U-value, Z-value, p-value is calculated by Mann-Whitney U test ^*^Total cost involved both cost incurred for blood group discrepancy resolution (INR 1730) and number of units phenotyped for M antigen ^**^Number of working days needed to make a unit available for the patient (Though the number of hours required to resolve the case was <24hrs) ^***^Number of days the surgery was shifted (even though the blood unit was ready, but the OT slot available for the surgeon might be weekly once/twice) ^a^Cost for routine blood grouping (INR 150) and crossmatching (INR 150) amounts to INR 300

	Patients with anti-M^1^ Median (IQR)	Routine cases^2^ Median (IQR)	U- value^3^	Z-value^3^	p-value^3^
Duration for Blood grouping (in minutes)	480 (477-522)	34 (32-40.5)	0	-5.20	<0.05
Number of units phenotyped to find a M-negative unit	1 in 13 (20 units from 247 units)	NA			
Turnaround time to find a compatible unit (in minutes )	1440 (945-1875)	72 (68-76.25)	0	-5.20	<0.05
Additional cost per patient in INR (total cost incurred/no. of patients)^*^	3530 (3530-4730)	zero^a^	0	-4.98	<0.05
Delay in schedule transfusion (in days)^**^	2	zero	0	-4.37	<0.05
Delay in scheduled OT (in days)^***^	2 (2-7.25)	NA			

For these patients, M-antigen negative was searched and almost 247 units were phenotyped. Twenty units were M antigen negative, which means almost one unit among 10-14 units. The turnaround time to make the blood unit available for a patient was significantly higher (p<0.05) with a median of 24 (IQR: 15.75-31.25) hours. Hence, the transfusion schedule was delayed by almost two days. There was no demand for blood transfusion in seven cases, while in the rest eight cases, the surgical procedure was delayed by two (range two to eight) days. One patient (Case 13) required an M-negative PRC unit, but despite screening all 46 available B-positive units, a suitable match was not found. As a result, the patient was referred to another center, further delaying surgery. The difference between impact of anti-M in surgical and medical cases that required transfusions is depicted in Appendix 2.

In one case, anti-M with an IgG component and a titer of 1:16 was detected during pre-transfusion testing in an expectant mother who had been scheduled for lower segment caesarean section due to intra-uterine growth restriction with abnormal fetal Doppler. Following surgery, the neonate’s blood group was found to be O Rh D positive, similar to the mother’s, but M positive with a positive direct antiglobulin test. However, the baby was born with a birth weight of 1.73 kg and had respiratory distress, necessitating neonatal intensive care respiratory support. Hyperbilirubinemia developed at 24 hours of life, requiring intensive phototherapy for 48 hours. The newborn was discharged in stable condition on the 15th day of life.

The additional cost incurred by the blood center for resolving anti-M cases and securing compatible PRC units was significantly higher (p<0.05) at about INR 3530 (IQR: 3530-4730), excluding expenses related to advanced IH workup such as enzyme-treated cells and select cell panels.

Transfusion outcomes

M antigen-negative PRC units were successfully transfused to 11 patients with clinically significant anti-M. No transfusion reactions were observed. However, the prolonged time required to locate suitable blood units highlighted the logistical challenges of managing such cases, particularly in resource-limited settings.

## Discussion

Anti-M and its impact on transfusion management

While anti-M antibodies-predominantly of the IgM class-are generally considered clinically insignificant, our findings challenge this assumption by highlighting their practical impact on transfusion services. In this study, the presence of anti-M significantly affected pre-transfusion testing and logistics, often causing delays in transfusion turnaround times and surgical procedures.

Anti-M-associated blood group discrepancies required additional immunohematological workups, with a median resolution time of eight hours. For transfusion-dependent patients, sourcing M antigen-negative PRC units proved difficult due to the high prevalence of the M antigen in the Indian population (~88%). Statistically, one in 10 donor units is expected to be M-negative [1]. While this yield was generally observed, a notable exception occurred when 48 units were screened with no M-negative unit found, illustrating how probability does not always align with clinical availability.

This unpredictability complicates transfusion planning, especially in time-sensitive or resource-limited settings. The paradox lies in anti-M’s rare clinical relevance but disproportionately high logistical burden when significant. This necessitates labor-intensive screening protocols and robust donor inventories - resources often unavailable in many transfusion centers across India [10].

In our study, the cost of securing a compatible PRBC unit increased nearly 10-fold, from approximately INR 300 to INR 3,000. Surgical procedures were delayed by two to eight days, resulting in prolonged hospital stays and increased indirect costs for patients. Delays in securing compatible units also extended transfusion turnaround times [11]. Previous reports noted a median surgical delay of 12 hours (ranging from one to 168 hours) due to incomplete pre-transfusion workups [12].

Pei et al. suggested that universal preadmission type-and-screen testing-performed six to 28 days prior to surgery-can identify alloantibody-positive patients and help mitigate these delays [13]. These findings underscore the need for preemptive typing protocols, regional antigen-negative donor registries, and greater inter-institutional collaboration to optimize rare antibody management and avoid treatment delays.

Prevalence and clinical significance of anti-M

Over a two-year period, we identified 24 cases of anti-M antibodies - four in blood donors and 20 in patients - across all age groups. Among blood donors, anti-M was the second most common irregular antibody, following anti-H in the Bombay phenotype. This observation aligns with findings from Northern India [6].

Previous studies report anti-M prevalence in Indian donors ranging from 0.03% to 0.05% [6,14]. In contrast, our study found a lower prevalence of four (0.01%) in 28,620 donors, possibly due to differences in study design or population characteristics. Among patients, anti-M accounted for 10.3% of all detected irregular antibodies, aligning with international case reports citing prevalence rates between 2.9% and 14.26% [15,16].

The clinical significance of anti-M is closely related to its thermal amplitude. Cold-reacting IgM anti-M is typically benign; however, IgG or mixed IgM+IgG antibodies can cause HTRs and HDFN [17]. In our cohort, 22 (92%) of anti-M cases were clinically significant, showing reactivity at 37°C or in the AHG phase.

Twelve of our anti-M-positive patients were female, with eight of reproductive age (15-49 years). The presence of IgG anti-M in such cases poses a risk of HDFN, warranting close fetal monitoring. This may include serial ∆OD450 measurements via amniotic fluid analysis (Liley curve) and middle cerebral artery (MCA) Doppler assessments [3]. High-titer anti-M has also been associated with neonatal red cell aplasia and inhibition of erythroid progenitor proliferation [18,19]. In one notable case, an M-positive neonate developed mild HDFN, characterized by hyperbilirubinemia requiring phototherapy but without anemia. The newborn also had a low birth weight and respiratory distress, clinical features consistent with anti-M-mediated HDFN [20].

Interestingly, anti-M is frequently detected in pediatric patients in Polish, Indian, and Japanese studies, particularly under the age of five [21]. However, we identified only two pediatric cases, neither associated with concurrent infections. Furthermore, while anti-M in Polish patients is predominantly IgM, Indian patients more commonly exhibit the clinically significant IgG form [7,22]. Our findings reflect this trend, with both IgG and IgM components observed in donors and patients alike, consistent with a Chinese study by Wang et al. [16].

Genetic, environmental, and immunological factors likely contribute to this variability. Chronic infections, repeated antigenic exposures, and differential HLA-linked immune regulation may increase the risk of alloimmunization and influence antibody characteristics in Indian patients [23]. A relatively higher prevalence of anti-M antibodies was observed among individuals with blood group O (12 out of 24) in our study, despite the absence of established evidence linking anti-M antibodies to specific ABO blood groups. This finding may be attributed to the fact that blood group O is the most prevalent in our region [24]. Given the comparatively higher frequency of the Bombay phenotype in our population [25], it is essential to utilize anti-H lectin to rule out this rare blood group. However, this additional step contributes to increased costs and potential delays in the blood typing process

Unusual anti-M reactivity and possible genetic variants

In two cases (4 and 5), M antigen reactivity persisted despite papain treatment, which typically eliminates M antigen expression. This unusual resistance suggests the presence of variant M antigens or underlying genetic alterations. Variations in MN antigen expression can result from hybrid gene formations, such as GPYA-B-A, which have been linked to MN typing discrepancies [26].

The involvement of a third glycophorin gene, GYPE, further increases the likelihood of recombination and hybrid molecule formation. For instance, the GYP(B-E-B) hybrid allele encodes a GP(E-B) molecule that expresses a trypsin-resistant M antigen [27,28]. Such hybrids complicate serological identification, particularly when commercial monoclonal anti-M reagents are used. These reagents may cross-react with atypical glycophorin variants, while polyclonal anti-M, due to its epitope specificity, may fail to detect certain modified antigens [29].

In our study, genotyping was not available. However, the lack of reactivity to polyclonal and autologous anti-M, alongside positive results with monoclonal reagents, supports the hypothesis of glycophorin variants. Additionally, anti-M is known to exhibit a dosage effect, showing stronger reactivity with M+N− cells than with M+N+ cells - a pattern we observed across all cases. This phenomenon complicates the identification of weak anti-M antibodies and may lead to underdiagnosis.

Our study is subject to several limitations. The retrospective design may have led to underreporting, especially in patients with weak or low-titre anti-M. Furthermore, the absence of molecular testing limited our ability to confirm the genetic basis of the suspected antigen variants. Future prospective multicenter studies incorporating genotypic analysis and larger sample sizes are essential to further elucidate the epidemiology, clinical significance, and molecular underpinnings of anti-M antibodies in diverse population.

## Conclusions

Our study underscores the importance of recognizing anti-M as a clinically relevant antibody with significant impact on transfusion services. The high prevalence of clinically significant anti-M, the associated delays in transfusion schedule and surgical procedures, and the challenges in sourcing M-negative units highlight the need for proactive donor screening and improved transfusion strategies. Establishing a rare donor registry and enhancing IH laboratory capabilities could help mitigate these challenges. Further research, particularly involving molecular and population-based studies, is warranted to better understand the genetic and serologic diversity of the MNS blood group system and its clinical impact.

## Appendices

Appendix 1

**Table 3:** Demography details and characteristics of anti-M in Patient Population BG: Blood Group, CVA: Cerebro Vascular Accident, DCLD: Decompensated Chronic Liver Disease, DIC: Disseminated Intravascular Coagulation, GA: Gestational Age, IUGR: Intra-uterine growth restriction, IT: Inter-trochanteric, MS: Mitral stenosis, Neg: Negative, No.: Number of, ORIF: Open Reduction and Internal Fixation, Pos: Positive, RHD: Rheumatic heart disease, SCD: Sickle cell Disease, TAT: Turnaround time, TH: Transfusion History

Case-No	Age	Sex	Diagnosis	Indication	TH	BG	Characteristics of anti-M	M phenotype	Requirement of PRC transfusion	No. of M Neg Units found and total no. of bags typed	Effect of anti-M on Hospitalization
5	4	M	Right Inguinal Hernia	Laproscopic Herniotomy	Nil	B Rh D Pos	IgM + IgG	4+, resistant to enzyme	No	'-'	Surgery postponed by 07 days
6	66	M	Hydrocele and cholelithiasis	Laproscopic cholecystectomy	Nil	O Rh D Pos	IgM	1+, resistant to enzyme	No	'-'	'-'
7	28	M	Right sided shaft of femur fracture	Implant Removal	Nil	A Rh D Pos	IgM + IgG	Neg	Yes	2 in 18 bags	Surgery postponed by 02 days TAT:7 HOURS
8	34	F	Pre auricular synus of right ear	Fistulectomy	Nil	A Rh D Pos	IgM + IgG	Neg	No	'-'	'-'
9	80	F	Carcinoma tongue	Surgical biopsy	Nil	O Rh D Pos	IgM + IgG	Neg	No	'-'	'-'
10	30	F	Cholelithiasis	Laproscopic cholecystectomy	Nil	O Rh D Pos	IgM + IgG	Neg	No	'-'	'-'
11	23	F	POD 20 LSCS with abdominal tuberculosis	Secondary suturing	Nil	A Rh D Pos	IgM + IgG	Neg	No	'-'	Surgery postponed by 01 days
12	46	F	Abnormal uterine bleed unspecified	Abdominal Hysterectomy with bilateral salpingectomy with left oopherectomy	Nil	O Rh D Pos	IgM + IgG	Neg	Yes	2 in 48 bags	Surgery postponed by 08 days TAT: 32 HOURS
13	33	F	RHD with severe calcific MS	Mitral Valve Replacement	4 units PRC	B Rh D Pos	IgG	Neg	Not found	0 in 46 bags	Surgery postponed by 05 days REFERRED TO OTHER HOSPITAL
14	39	F	Ureteric calculi	Uretrorenoscopy	Nil	A Rh D Pos	IgM + IgG	Neg	No	'-'	'-'
15	53	F	Pituitary Macroadenoma	Transnasal and Transpenoidal decompression of the tumour	Nil	B Rh D Pos	IgM + IgG	Neg	Yes	1in 18 bags	Surgery postponed by 02 days TAT:22 hours
16	24	F	Primigravida (36 weeks GA) with hypothyroidims with preeclampsia with IUGR and abnormal Doppler changes	Caeserian section	NIL	O Rh D Pos	IgM + IgG	Neg	No	2 in 8 bags	TAT:17 HOURS
17	65	F	CVA	External Ventricular Drainage	NIL	AB Rh D Pos	IgM + IgG	Neg	Yes	1 in 7 bags	Surgery postponed by 02 days TAT:32 HOURS
18	71	F	Left distal femur fracture	ORIF with plating	Yes	O Rh D Pos	IgG	Neg	Yes	2 in 12 bags	Surgery postponed by 02 days TAT:17 HOURS
19	69	M	Right IT Fracture	Proximal Femoral Nail for IT fracture	Nil	B Rh D Pos	IgG	Neg	Yes	2 in 12 bags	Surgery postponed by 07 days TAT: 32 HOURS
20	35	M	SCD with acute hepatitis and DIC	Anaemia	Yes	A Rh D Pos	IgG	Neg	Yes	2 in 31 bags	TAT:29 HOURS
21	47	F	DCLD	Anaemia	Yes	O Rh D Pos	IgM + IgG	Neg	Yes	1 in 3 bags	TAT:12 HOURS
22	49	M	Cirrhosis of liver	Anaemia	Nil	B Rh D Pos	IgG	Neg	Yes	2 in 12 bags	TAT:26 HOURS
23	70	M	CVA	Anaemia	Yes	O Rh D Pos	IgG	Neg	Yes	2 in 12 bags	TAT:27 HOURS
24	7	M	Acute Pancreatitis	Anaemia	Nil	O Rh D Pos	IgM + IgG	Neg	Yes	1 in 20 bags	TAT:12 HOURS

Appendix 2

**Table 4:** Impact of anti-M on medical cases vs surgical cases who required transfusions *Number of working days needed to make a unit available for the patient (Though the number of hours required to resolve the case was <24hrs) **Number of days the surgery was shifted (even though the blood unit was ready, but the OT slot available for the surgeon might be weekly once/twice)

	Medical (n=05) Median(Range)	Surgical (n=08) Median(Range)
Blood group discrepancy resolution duration (in hours)	8 (7-9)	8 (7-9)
Number of units phenotyped to find a M-negative unit	1 in 10 bags (8 units among 78 bags)	1 in 14 units (12 units among 169 units)
Turn around time to find a compatible unit (in hours)	26 (12-28)	22 (17-34)
Delay in schedule transfusion (in days)*	2 (1-2)	2 (1-5)
Delay in scheduled OT (in days)**	NA	2 (2-8)
